# Whole-genome analysis reveals possible sources of ALV-J infection in an anyi tile-like gray chicken flock

**DOI:** 10.1016/j.psj.2022.101764

**Published:** 2022-01-30

**Authors:** Hai-Qin Li, Fan-fan Zhang, Longfei Chen, Qun Yang, Yan-Bing Zeng, Jia Tan, Guang-Hua Fu, Qiu-Ling Fu, Rong-Chang Liu, Yu Huang, Qi Su, Mei-Fang Tan, Zhao-Feng Kang

**Affiliations:** ⁎ Institute of Animal Husbandry and Veterinary Medicine, Jiangxi Academy of Agricultural Sciences, Nanchang 330200, China; † College of Animal Medicine, Shandong Agricultural University, Tai'an, Shandong, 271000, China; ‡ Institute of Animal Husbandry and Veterinary Medicine, Fujian Academy of Agricultural Sciences, Fuzhou 350013, China

**Keywords:** avian leukosis virus, subgroup J, local chickens, epidemiology, whole genome

## Abstract

Avian leukosis virus (**ALV**) induces multiple tumors in chicken and is still prevalent in a lot of local flocks in China. In this study, we analyzed the ALV infection status in an Anyi tile-like gray chicken flock by DF1-cells isolation, virus identification, and genome sequencing. Results showed a 29% (29/100) ALV positive rate in this flock. Homology analysis based on *env* genes illustrated that all these stains belong to subgroup J (92–100% identities) and can be further divided into 5 batches, suggesting a higher diversity of ALV-J within the same flock. The whole-genome analysis of representative stains from each batch confirmed the close relationship between these isolated strains with previously reported strains from different regions (Guangxi, Shandong, and Heilongjiang), revealing the enrichment of different strains in Anyi tile-like grey chickens. This study provides the epidemiological data of ALV-J in a special chicken flock and a reference for the further eradication of ALV in China.

## INTRODUCTION

Avian leukosis virus (**ALV**) belongs to the genus *alpharetrovirus* and induces leukosis and tumor in poultry (Payne et al., 1991; Kaleta et al., 2000). Up to now, 7 different ALV subgroups (A, B, C, D, E, J, and K) have been reported from chickens (Bai et al., 1995; Dong et al., 2015). Among them, ALV-J is the most pathogenic, which leads to myelocytoma, hemangioma, and multiple other malignant tumors (Weiss and Vogt, 2011). In China, ALV-J was first isolated in 1999 and then circulated for a long time. After the Nationwide Eradication Program launched in 2008 (Du et al., 1999; Gao et al., 2010), the prevalence of ALV-J decreased rapidly, especially in commercial broiler and layer chickens (Feng and Zhang, 2016). However, a lot of studies suggested that ALV-J is still circulating in most local chicken flocks in China (Su et al., 2018a), and a lot mutant strains have been bred in the long evolution, posing a potential huge threat to the poultry industry (Su et al., 2018b, 2020; Deng et al., 2021).

Previous study suggested that local chicken flocks may serve as reservoirs for different ALV strains and provide a hotbed for virus evolution and mutation, but how the virus flows in and out remains unclear (Li et al., 2021). Since *env* gene is the most easily mutated gene of ALV, especially subtype J, the molecular epidemiological analysis should not only focus on this gene but also pay close attention to the whole genome, to produce more solid conclusions. To this end, we analyzed the ALV infection status in an Anyi tile-like gray chicken flock and sequenced the whole genome to explore the possible source of the virus.

## MATERIALS AND METHODS

### Background of the Chicken Flock

Anyi tile-like gray chicken is an unique local chicken of Anyi County, Jiangxi Province, China (Supplementary Figure 1). Since late 2018, AL-like tumor diseases have suddenly appeared in those chickens (Li et al., 2021). The average age of suspected AL onset was around 135-day-old, and the symptoms last for 30 to 37 wk with an obvious peak period about 32-wk-old. Ten thousand chickens were raised in the chicken farm analyzed in this study. According to one percent of the breeding scale, plasma samples were randomly collected from 100 chickens of this farm and stored in blood collection vessel containing heparin sodium for further analysis.

### Virus Isolation and Identification

Virus identifications were performed in DF-1 chicken fibroblast cell line (American Type Culture Collection, Manassas, VA) maintained in our laboratory. The DF-1 cells were cultured in Dulbecco's modified Eagle's medium (**DMEM**; Invitrogen) with 12% fetal bovine serum (**FBS**; Invitrogen) at 37°C in a 5% CO_2_ incubator. Lymphocytes from the plasma samples were incubated on DF-1 cells in 24-well culture plates after centrifugation at 1,500 × *g* for 2 min. Uninfected DF-1 cells were served as the negative control. The culture supernatant was harvested 7 d later, and the cells were passaged to the next generation. After 3 blind passages of infected cells, the cell supernatants and cell samples were harvested and then stored at −80°C until analysis. After 3 freeze-thaw cycles, the supernatant samples from each well (described previously, Su et al., 2018a) were examined for the presence of ALV group-specific P27 antigen using the ALV P27 Antigen Test Kit (IDEXX; Yuanheng Laboratories, China) as described previously (Su et al., 2018a).

### Genomic DNA Extraction and Subgroups Identification

DNA was isolated from ALV-positive cells using a commercial kit (Bio-Tek, Norcross, GA), and total DNA was resuspended in 12.25 µL of DNase-, RNase-, and proteinase-free water. For subgroup identification by polymerase chain reaction (**PCR**), positively infected DF-1 cells were selected as a template for subgroup-specific amplification (using the env primers shown in Table 1). Uninfected DF-1 cells were served as negative control.

**Table 1:** Primers used in this study.

Primer^a^	Sequence	Fragment size(bp)
env-F	5′-GATGAGGCGAGCCCTCTCTTTG-3′	2,300
env-R	5′-TGTTGGGAGGTAAAATGGCGT-3′	
A-F	5′-GAGATTGTCTGCAGGGCCTAGGGCT-3′	2,715
A-R	5′-TGGCAGCAAGGGTGTCTTCTCCG-3′	
B-F	5′-CACCACATTGGTGTGCACCTGGGT-3′	2,778
B-R	5′-GAAGGGGCCACTGGTCAATCCACA-3′	
C-F	5′-GAGGTGACTAAGAAAGATGAGGCGA-3′	2,124
C-R	5′-CATCTCCCCCTCCCTATGCGAAAGC-3′	
D-F	5′-ATTGGAGCAGTGTAAGCAGTACG-3′	1,148
D-R	5′-CGTTTATGACGCTTCCATGCTTG-3′	

a F and R represent upstream and downstream primers, respectively.

### Whole-Genome Sequencing

The whole genome of the above-isolated strains was amplified by PCR using genomic DNA extracted from infected DF-1 cells as a template with Premix LA Taq polymerase (TaKaRa, Dalian, China) in a 50-μL reaction containing 4 μL of dNTP mixture (TaKaRa), 5 μL of 10 × PCR buffer (TaKaRa), 1 μL of Taq polymerase (TaKaRa), 2 μL of DNA solution, 1 μL of forward and reverse primers, and 36 μL of ddH_2_O. The primers and corresponding thermocycling profiles used in this study are designed in a previous study (Su et al., 2018a). The PCR products were purified by 1% agarose gel electrophoresis and then recycled by the Omega Gel Extraction Kit (Guangzhou, China). The purified products were then cloned into the pMD18-T vector (Transgen, China), and the resulting construct was used to transform *E. coli* DH5α cells (TaRaKa). Positive clones were sequenced by a commercial company (Shenggong, Shanghai, China), and each one was sequenced at least 3 times independently.

### Sequence Alignment and Analysis

Obtained sequences of the above isolates were assembled using DNAStar (version 7.0), and multiple sequence alignment was obtained using Clustal X (BioEdit version 7.0) and Blast (NCBI). Nucleotide and deduced amino acid sequence similarity searches were performed using MEGA (version 5.0) and online BLAST tool (https://blast.ncbi.nlm.nih.gov/Blast.cgi). The phylogenetic analysis was performed using the maximum likelihood method on MEGA 5.0. The sequences obtained in this study have been deposited in GenBank.

### Ethics Statement

The study protocol and all animal experiments were approved by the Animal Ethics Committee of the Institute of Animal Husbandry and Veterinary, Jiangxi Academy of Agricultural Science (2010-JXAAS-XM-01). All methods were performed following the relevant guidelines and regulations.

### Data Availability Statement

The data that support the findings of this study are available in Genbank. The reference numbers for *env* genes sequenced in this study is from MT262540-MT262568. The reference numbers for the whole genome are MU482451-MU482456.

## RESULTS

### Isolation and Identification of ALV

A total of 100 blood samples were analyzed by DF-1 cells virus isolation and Anti-P27 ELISA assay. Among them, 29 samples were determined as ALV positive and then used for subgroup classification by *env* gene sequencing (Supplementary Figure 2 and Table 1). The length of these *env* genes ranged from 1,677 bp to 1,707 bp, and all of them shared the highest identity with published ALV-J reference strains using the online BLAST program (https://blast.ncbi.nlm.nih.gov/Blast.cgi), suggesting they all belong to ALV-J (Supplementary Table 2).

### Env Gene Analysis Reveals the Diversity of ALV-J in a Singly Flock

**Figure 1:**
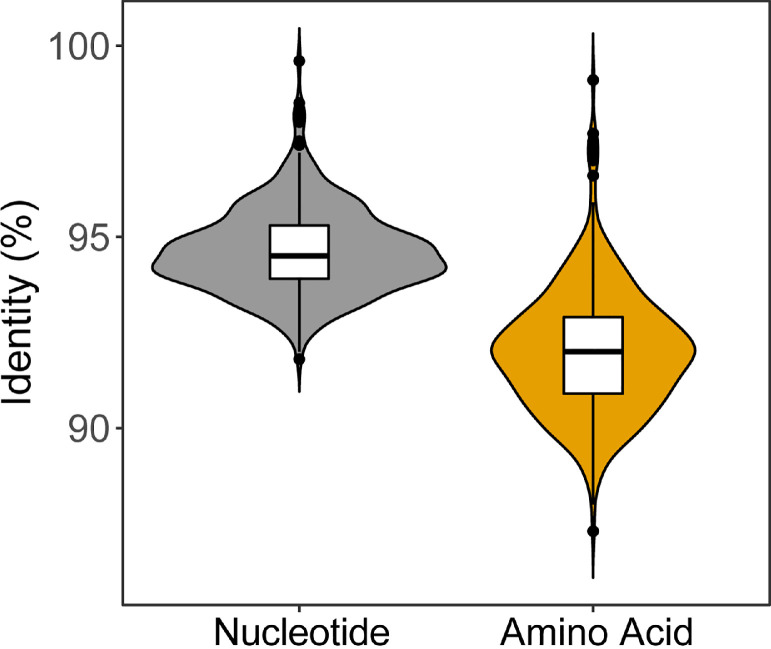
The nucleotide and amino acid sequence identity among the *env* gene of 29 ALV-J strains isolated in this study.

**Figure 2:**
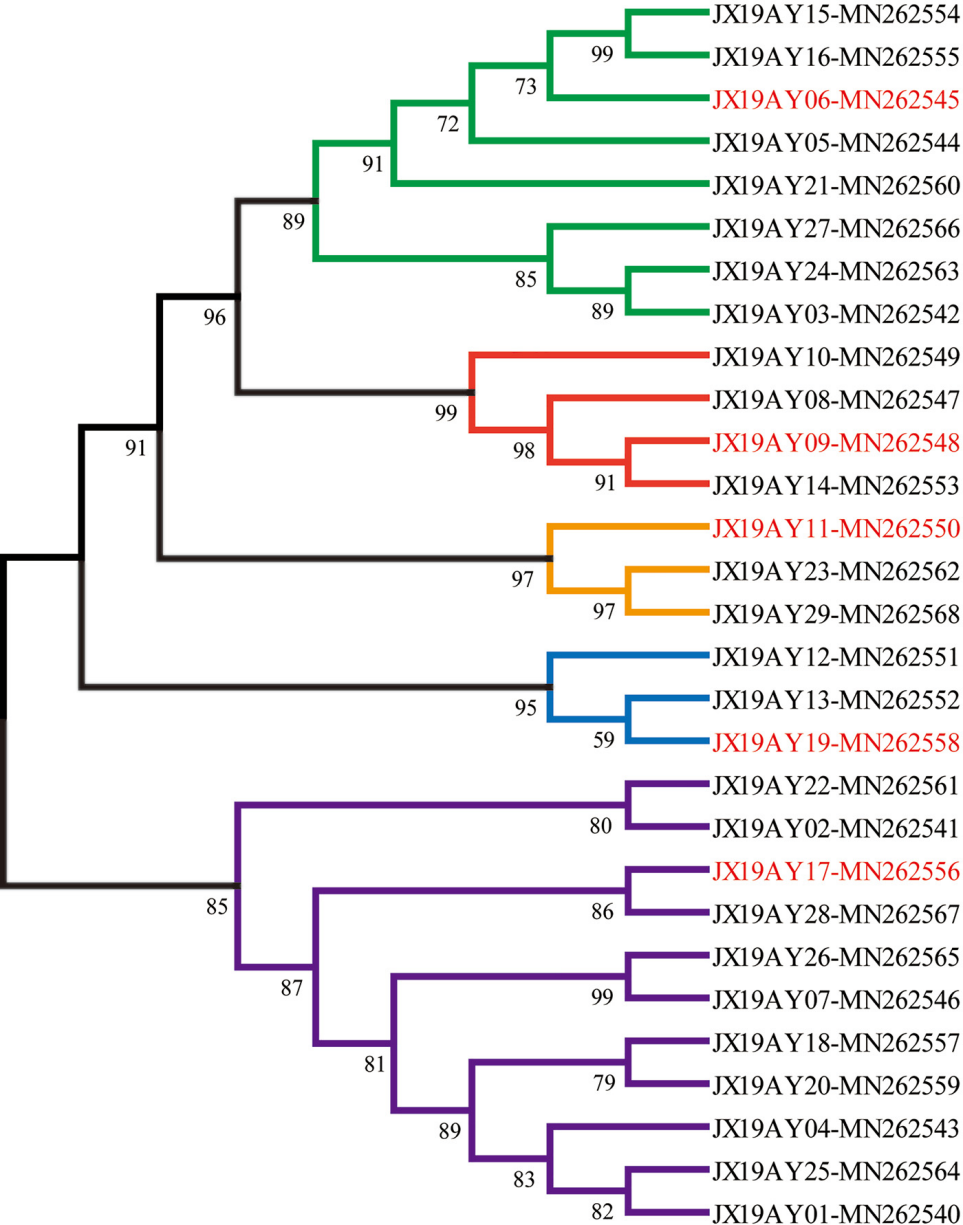
Phylogenetic tree based on the env gene sequences of 29 ALV strains isolated in this study. The tree was constructed by the maximum likelihood method with 1,000 bootstrap replicates using MEGA 5.0. The five representative strains used for whole genome sequencing were marked in red.

We further compared the *env* genes both of nucleotide and amino acid sequences using MEGA (version 5.0) to understand the molecular epidemiology of ALV-J in Anyi tile-like gray chickens. We found that the homology within these 29 ALV-J isolates is relatively low. The average homology of the nucleotide sequence is 94.6%, while the average homology of the amino acid sequence is only 92% (Figure 1, Supplementary Table 3). Phylogenetic analysis based on these *env* genes further grouped them into 5 groups (Figure 2), and each of them shared a relatively remote evolution relationship with others (Supplementary Table 3).

### Whole-Genome Analysis of Representative Strains

To find the possible source of ALV infection in the flock studied, we further sequenced the whole genome of 5 representative strains and analyzed them in detail. The representative strains were selected based on the phylogenetic analysis and marked in the evolution tree (Figure 2). After sequencing and assembly, the whole genomes of all these 5 strains were generated and then submitted into the Bankit (NCBI) with accession number from MU482451-MU482456. The length of them ranged from 7,605 bp to 7,633 bp under a genetic organization typical of replication-competent retroviruses lacking viral oncogenes (5’-LTR-leader-gag-pol-env-3’-LTR).

The genome diversity was confirmed again at the whole genome level, and these 5 strains shared relatively low identities from 93.6% to 95.5 with each other (Table 2). The difference was much larger in *env* genes, where there were only about 90% identities in the translated amino acid sequences (Table 2). As reported in previous studies, the *pol* and *gag* gene were conservative, and both of them in the ALV-J strains sequenced in this study exhibited around 97% identities (Table 2).

**Table 2:** Whole genome analysis of the representative strains.

Strains	JX19AY06	JX19AY09	JX19AY11	JX19AY17	JX19AY19
Whole genome					
JX19AY06		95.2	93.6	94.9	95.3
JX19AY09			95.5	95.4	95.1
JX19AY11				93.8	93.9
JX19AY17					94.5
JX19AY19					
*Pol*					
JX19AY06		97.2	97.9	98.4	97.0
JX19AY09	**98.8**		97.3	97.9	96.3
JX19AY11	**99.4**	**98.3**		98.9	97.2
JX19AY17	**99.4**	**98.6**	**99.3**		97.9
JX19AY19	**98.8**	**97.7**	**98.1**	**98.7**	
*Gag*					
JX19AY06		95.0	94.9	94.6	95.3
JX19AY09	**98.1**		96.6	96.3	95.9
JX19AY11	**97.8**	**98.0**		95.5	95.9
JX19AY17	**96.5**	**97.6**	**97.2**		95.5
JX19AY19	**98.3**	**98.1**	**98.1**	**97.0**	
*Env*					
JX19AY06		93.2	93.9	94.8	95.1
JX19AY09	**89.5**		98.2	93.3	92.6
JX19AY11	**91.1**	**91.5**		94.4	93.1
JX19AY17	**92.3**	**89.7**	**91.6**		93.4
JX19AY19	**92.0**	**90.0**	**90.9**	**89.5**	

The identities of amino acid sequence are in bold.

**Figure 3:**
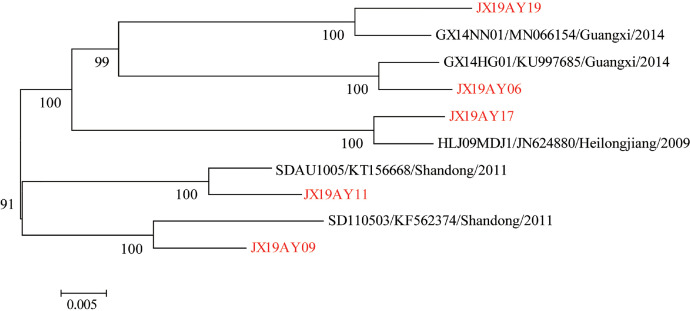
Phylogenetic tree based on the whole genome of five representative strains and corresponding most similar known strain from NCBI. The tree was constructed by the maximum likelihood method with 1,000 bootstrap replicates using MEGA 5.0. The five representative strains isolated in this study were marked in red.

For further identifying the potential source of ALV infection in the Anyi tile-like gray chickens, the online BLAST program (https://blast.ncbi.nlm.nih.gov/Blast.cgi) was used to determine their similarity with all the published ALV strains, and the information of the reference strain with the highest similarity was recorded for analysis. As shown in Table 3, the identities between these 5 ALV-J strains and corresponding the most similar known strain were from 98.41 to 99.08%. It was striking that those reference strains were isolated at different time points, from different regions of China and different chicken types (Table 3). Phylogenetic analysis based on the whole genome further confirmed the evolving relationship between ALV-J strains isolated in this study and corresponding the most similar known strains (Figure 3). The five representative strains were scattered on different branches, showing remote evolution relationships with others (Figure 3).

**Table 3:** ALV-J isolated in this study and one to one corresponding the most similar reference strain published in Genbank

Strains	Most similar known strain	Similarity (%)	Time	Location	Source
JX19AY06	GX14HG01-KU997685	99.03%	2014	Guangxi	Chicken
JX19AY09	SD110503-KF562374	98.41%	2011	Shandong	Chicken
JX19AY11	SDAU1005-KT156668	98.85%	2011	Shandong	Chicken
JX19AY17	HLJ09MDJ1-JN624880	99.08%	2009	Heilongjiang	Layer chicken
JX19AY19	GX14NN01-MN066154	98.70%	2014	Guangxi	Three yellow chicken

The geographic location of the isolates in China is accurate to the provinces.

## DISCUSSION

Recently, the ALV infections in Chinese local chickens were reported frequently (Su et al., 2018a), and many variants have been identified (Su et al., 2018b, 2020; Deng et al., 2021). However, almost all these studies analyzed the ALV infection status in yellow chickens, which is the most commercially valuable local chicken in China, but little in other small but highly biologically valuable and genetically diverse chicken flocks. Unlike other chicken flocks with mixed infection of ALV-A, ALV-B, ALV-J, and the newly identified ALV-K (Su et al., 2018a, 2019; Mao et al., 2020), the prevalent subgroup is ALV-J in Anyi tile-like gray chickens with a surprising positive rate of 29%. As a virus mainly spread vertically, such a high positive rate is sufficient to reflect that ALV-J has existed in this flock for a long time, revealing the important role of local chickens in the transmission route of ALV-J.

Previous studies have shown the genome diversity of ALV-J within certain flocks and demonstrated the existence of virus quasispecies (Meng et al., 2016). However, in this study, the identities between strains isolated from the same flock were very low based on the *env* genes, less than 95%, which has already broken through the category of quasi-species. The emergence of multiple different strains in the same chicken flock should be considered as the aggregation of strains from different sources like horizontal transmission or use of contaminated vaccines (Wang et al., 2018, 2021; Mao et al., 2020), demonstrating that small and easily overlooked local chicken flocks have become reservoirs for a variety of strains.

With the help of whole-genome analysis, we further determined the genome characteristics of these strains. The representative strains sequenced in this study exhibited greater genome differences at the whole genome level, which further confirmed that they may be from different sources. Through the online BLAST tool, we found the most similar known strain for these 5 representative strains, respectively, and noticed that they were isolated from different time points, locations, and even chicken breeds. It is worth noting that the above reference strains were isolated from Heilongjiang Province, Shandong Province, and Guangxi Province. They located in the Northeast, East, and Southwest of China, respectively, which are very far away from Jiangxi Province, where the chicken flock analyzed by this study are located. We don't know how these strains span such a large distance, but it is enough to reflect the widespread and prevalence of ALV strains in China.

The nationwide ALV eradication program has achieved great success in commercial broiler and layer chickens with almost no report about the outbreak of AL after 2013 (Wang et al., 2018), but this does not fully support that we can relax our vigilance against ALV. Especially after 2018, there has been another outbreak of myelocytomatosis of unknown origin in white feather broilers caused by several mutational ALV-J isolates in China, which have a lot of newly emerged genomic features that may be related to increased pathogenicity and tissue tropism (Zhou et al., 2019; Zhang et al., 2020). This study observed the enrichment of old strains in local chicken breeds, while whether this will cause the outflow of the virus strains needs further epidemiology analysis.

In conclusion, this study systematically observed the diversity of ALV strains in chicken flocks, confirmed the prevalence of multiple strains in the same chicken flock, and provided a reference for future epidemiological analysis about the ALV in China.

## Appendix Supplementary materials

**Supplementary Figure 1** Anyi Tile-Like Grey Chicken Image, image 1

**Supplementary Figure 2** Electrophoretic analysis of env gene of 29 isolates in this study Image, image 2

**Supplementary Table 1** The homology analysis among the env genes of 29 ALV-J strains isolated in this study. Image, application 3

**Supplementary Table 2** The most similar reference strains with 29 isolates in this study Image, application 4

Image, application 5

## Footnotes

Supplementary material associated with this article can be found in the online version at doi:10.1016/j.psj.2022.101764.
