# Supplementary figures and images for: Whole-genome analysis reveals possible sources of ALV-J infection in an anyi tile-like gray chicken flock
Source: Poult Sci. 2022 Jan 30;101(5):101764. doi: 10.1016/j.psj.2022.101764 (PMC8980333; doi:10.1016/j.psj.2022.101764)

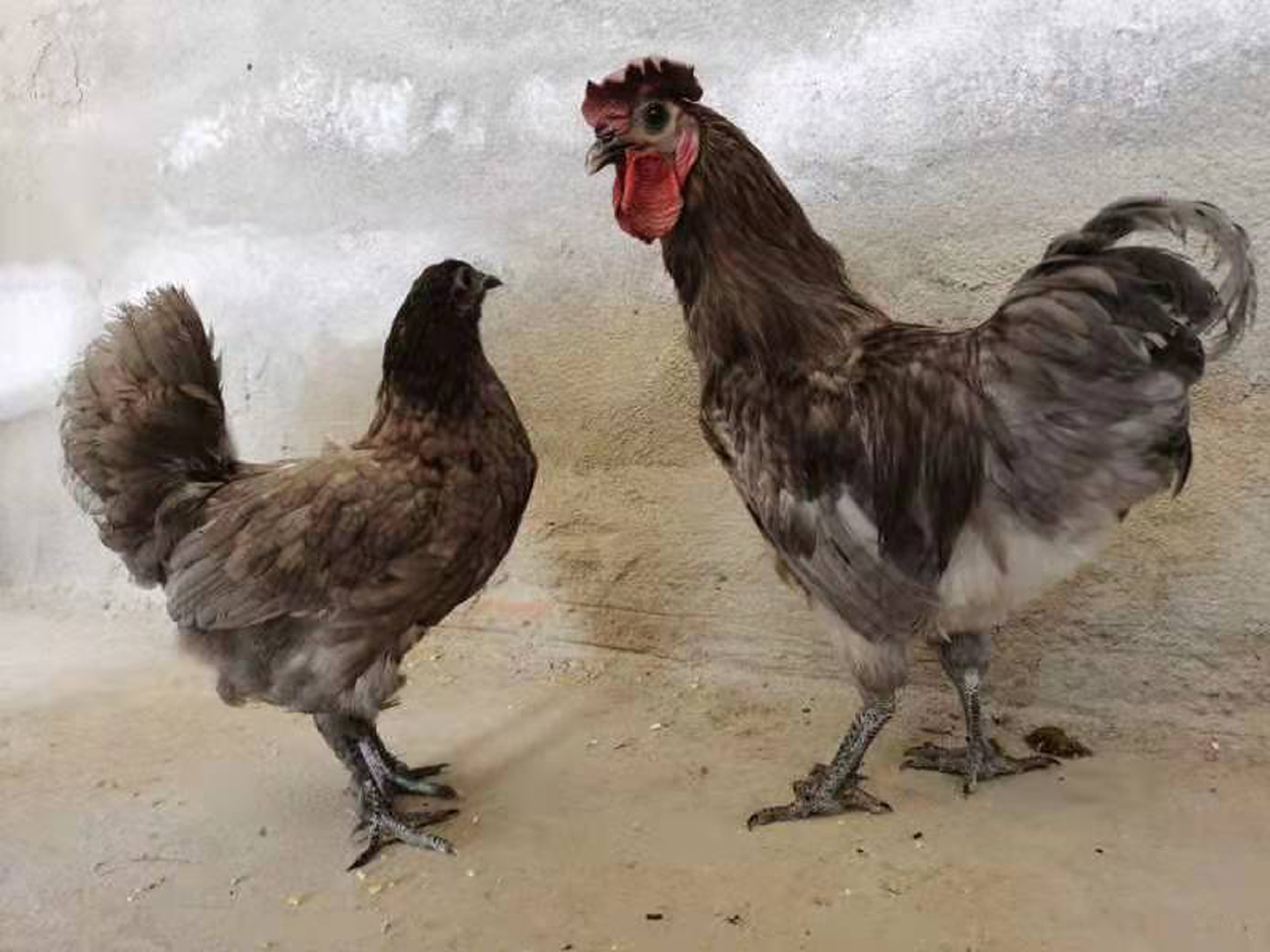

Supplement: Supplementary file 1 — Supplementary Figure 1 Anyi Tile-Like Grey Chicken [file mmc1.jpg]

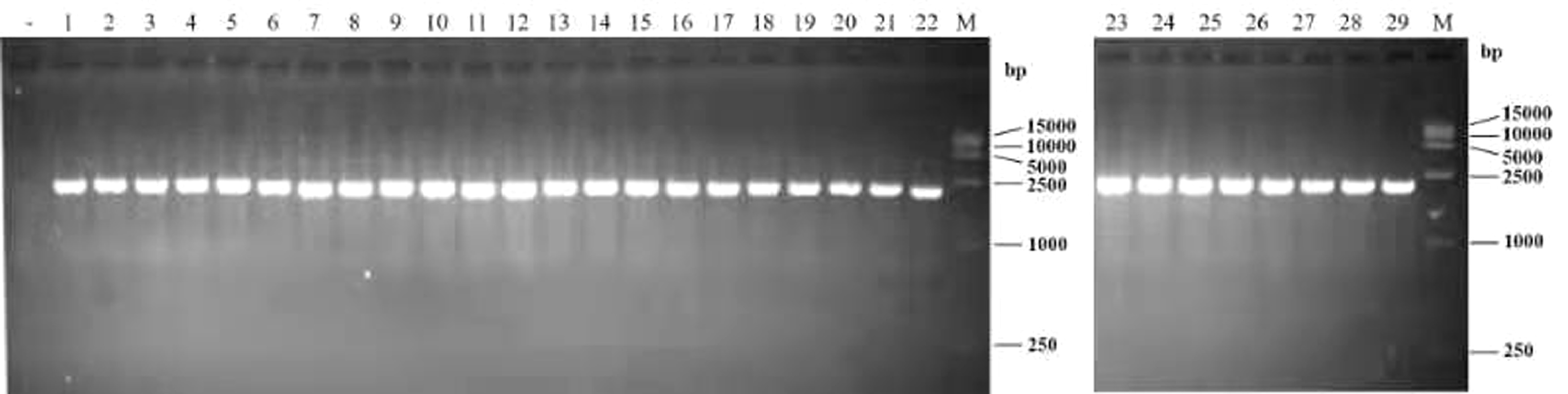

Supplement: Supplementary file 2 — Supplementary Figure 2 Electrophoretic analysis of env gene of 29 isolates in this study [file mmc2.jpg]
